# Causal association of metformin treatment with diverse immune-mediated inflammatory diseases: A Mendelian randomization analysis

**DOI:** 10.1097/MD.0000000000041400

**Published:** 2025-02-07

**Authors:** Zheng Liao, Chenguang Su, Jian Li, Jinlong Liu

**Affiliations:** a Department of Hepatobiliary Surgery, Affiliated Hospital of Chengde Medical University, Chengde, Hebei, China.

**Keywords:** causal effect, genome-wide association studies, immune-mediated inflammatory diseases, Mendelian, metformin

## Abstract

Metformin has been shown to possess immune-modulating and anti-inflammatory effects in various animal and clinical studies. It is believed to be effective in treating some immune-mediated inflammatory diseases (IMIDs). However, there remains ongoing debate regarding the extent to which metformin can reduce the risk of developing IMIDs. We used the data from genome-wide association studies to explore the causal relationship between metformin treatment and some IMIDs through the Mendelian randomization (MR) analysis. Additionally, sensitivity analyses were performed using the Cochran *Q*-test, MR-PRESSO and “leave-one-out” to confirm the robustness of our conclusions. The MR analysis indicated that metformin treatment could reduce the risk of rheumatoid arthritis (RA) (OR = 0.018, 95% CI: 1.33 × 10^−3^–0.233, *P* = .002), multiple sclerosis (MS) (OR = 0.966, 95% CI: 0.936–0.997, *P* = .030) and primary sclerosing cholangitis (PSC) (OR = 6.82 × 10^−4^, 95% CI: 7.83 × 10^−6^–5.93 × 10^−2^, *P* = .001). But metformin treatment is not significantly associated with the risk of Crohn disease (OR = 0.994, 95% CI: 0.979–1.009, *P* = .431), ulcerative colitis (UC) (OR = 0.987, 95% CI: 0.965–1.009, *P* = .234), systemic lupus erythematosus (SLE) (OR = 164.373, 95% CI: 0.158–1.71 × 10^5^, *P* = .150), autoimmune hepatitis (AIH) (OR = 2.909, 95% CI: 4.58 × 10^−3^–1.85 × 10^3^, *P* = .746) and primary biliary cholangitis (PBC) (OR = 0.055, 95% CI: 1.44 × 10^−3^–2.112, *P* = .119). Due to the heterogeneity of the data from UC, SLE, MS, and PBC, we adjusted them. After adjustment, there is no change in the results for UC, SLE, MS, and PBC. The findings of this study support metformin treatment may reduce the risk of RA, MS, and PSC.

## 1. Introduction

immune-mediated inflammatory diseases (IMIDs) include over 100 different diseases, such as inflammatory bowel disease (IBD), rheumatoid arthritis (RA), skin inflammation, and connective tissue disease.^[1]^ This heterogeneous group of apparently unrelated conditions involving common inflammatory pathways and pathogenic mechanisms. Patients with 1 IMID are at an increased risk of developing another type.^[2]^ For instance, individuals with RA have a significantly increased risk of developing IBD.^[3]^ Consequently, the treatment of IMIDs is very important. Metformin is recognized for its ability to reduce inflammation and inhibit autoimmune inflammation, which makes it an attractive exploratory drug.

Metformin is a glucose-lowering agent primarily to treat type 2 diabetes mellitus (T2DM). It has become the first-line medication for individuals newly diagnosed with T2DM.^[4]^ Metformin also has the effects of the immune-modulating and anti-inflammatory. The effects of anti-inflammatory can operate through both AMPK-dependent and AMPK-independent mechanisms, as demonstrated in various interventional studies using rodent models of obesity and T2DM, as well as in vitro and ex vivo experiments involving several immune cell types.^[5,6]^ Metformin has been reported to exhibit various immunomodulatory effects, including enhancing immunosuppressive capacity in certain autoimmune inflammatory diseases,^[7,8]^ modulating immune responses to infections, and augmenting antitumor immunity.^[9,10]^ Some animal studies also have shown that metformin can treat inflammatory bowel disease, multiple sclerosis (MS) and autoimmune diseases.^[11–13]^ In the collagen antibody-induced arthritis murine model, metformin attenuated arthritis scores, bone destruction, serum levels of the pro-inflammatory cytokines.^[13]^ In RA, metformin can reduce the joint inflammation and destruction in human cell culture.^[14]^ And metformin is also effective in the treatment of systemic lupus erythematosus (SLE) and MS.^[15,16]^ However, some studies found no association between metformin treatment and some autoimmune disease.^[17,18]^

Mendelian randomization (MR) uses genetic variants as instrumental variables (IVs) to evaluate the causal effects of exposure factors on outcomes. This approach can mitigate the influence of confounding factors and reverse causation and the results have been convincing. Therefore, this study employed a 2-sample MR to investigate the relationship investigate metformin and various IMIDs.

## 2. Methods

### 2.1. Study design

**Figure 1.**
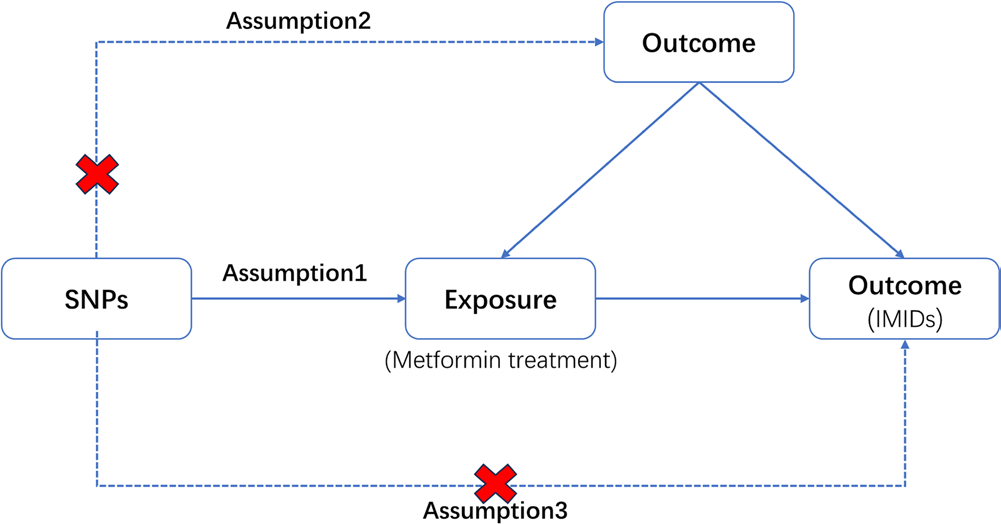
Flowchart of Mendelian randomization analysis in this study. IMIDs = immune-mediated inflammatory diseases, MR = Mendelian randomization; SNP = single-nucleotide polymorphism.

The workflow of the study is illustrated in Figure 1. We selected metformin as the exposure and some IMIDs (RA, Crohn disease [CD], ulcerative colitis [UC], SLE, MS, autoimmune hepatitis, primary biliary cholangitis, and primary sclerosing cholangitis [PSC]) as outcomes to conduct a forward MR analysis. The 2-sample MR applied in this study was based on genetic data obtained from publicly available genome-wide association studies (GWAS), which satisfied 3 basic assumptions^[19]^: First, the single-nucleotide polymorphisms (SNPs) selected as IVs should have a high degree of correlation with the exposure; Secondly, IVs must be independent of confounders; Finally, IVs should be associated with the outcome solely through metformin and not through alternative pathways.

### 2.2. Data sources

The exposure data set of metformin was obtained from publicly available GWAS data (https://gwas.mrcieu.ac.uk/). It included 462,933 Europeans individuals (11,552 cases and 451,381 controls) and 9,851,867 SNPs (GWAS ID: ukb-b-14609).^[20]^ The outcome data were also taken from the IEU Open GWAS project, with the data for RA (GWAS ID: ieu-a-832)^[21]^ comprised 58,284 samples (14,361 cases and 43,923 controls) and 8,747,963 SNPs, CD (GWAS ID: ukb-a-552)^[22]^ comprised 337,199 samples (732 cases and 336,467 controls) and 10,894,596 SNPs, UC (GWAS ID: ukb-b-19386)^[23]^ comprised 463,010 samples (1987 cases and 461,023 controls) and 9,851,867 SNPs, SLE (GWAS ID: ebi-a-GCST90018917)^[24]^ comprised 482,911 samples (647 cases and 482,264 controls) and 24,198,877 SNPs, MS (GWAS ID: ukb-b-17670)^[25]^ comprised 462,933 samples (1679 cases and 461,254 controls) and 9,851,867 SNPs, Autoimmune hepatitis (GWAS ID: ebi-a-GCST90018785)^[24]^ comprised 485,234 samples (821 cases and 484,413 controls) and 24,198,482 SNPs, primary biliary cholangitis (GWAS ID: ebi-a-GCST90061440)^[26]^ comprised 24,510 samples (8021 cases and 16,489 controls) and 5,004,018 SNPs, and PSC (GWAS ID: ieu-a-1112)^[27]^ comprised 14,890 samples (2871 cases and 12,019 controls) and 7,891,603 SNPs. The subjects of all datasets are European population. The details of the data source and definition are listed in Table 1.

**Table 1:** Characteristics of dataset used for MR analysis.

Item	GWAS ID	Year	Case (n)	Control (n)	Sample size (n)	Population	SNPs (n)
Metformin	ukb-b-14609	2018	11,552	451,381	462,933	European	9,851,867
RA	ieu-a-832	2014	14,361	43,923	58,284	European	8747,963
CD	ukb-a-552	2017	732	336,467	337,199	European	10,894,596
UC	ukb-b-19386	2018	1987	461,023	463,010	European	9851,867
SLE	ebi-a-GCST90018917	2021	657	482,264	482,911	European	24,198,877
MS	ukb-b-17670	2018	1679	461,254	462,933	European	9,851,867
AIH	ebi-a-GCST90018785	2021	821	484,413	485,234	European	24,198,482
PBC	ebi-a-GCST90061440	2021	8021	16,489	24,510	European	5,004,018
PSC	ieu-a-1112	2017	2871	12,019	14,890	European	7,891,603

Abbreviations: AIH = Autoimmune hepatitis, CD = Crohn disease, MR = Mendelian randomization, MS = multiple sclerosis, PBC = Primary biliary cholangitis, PSC = Primary sclerosing cholangitis, RA = rheumatoid arthritis, SLE = Systemic lupus erythematosus, SNPs = single-nucleotide polymorphisms, UC = ulcerative colitis.

### 2.3. SNPs selection

The SNPs selected as IVs conformed to the 3 principles described above. And we screened genome-wide for independent SNPs that were strongly associated (*P *< 5 × 10^−8^) with both the exposure and the outcome. The “TwoSampleMR” R package (version 0.5.8) (https://github.com/MRCIEU/TwoSampleMR)^[28]^ was used to delineate the screening thresholds for linkage disequilibrium (*r*^2^ < 0.001 and a window size of 10,000 kb) to exclude SNPs exhibiting high linkage disequilibrium. To ensure the consistent effect of IVs alleles across different databases regarding exposure and outcome, harmonization was performed. To eliminate the “weak instruments” (*F*-statistic < 10), we calculated the *F*-statistic for each SNP using the formula *F*=β^2^/SE^2^.^[29]^ β represents the estimated genetic effect on exposure, and SE represents standard error of β. Furthermore, to address potential bias due to confounding factors, we visited the PhenoScannerV2 GWAS database (http://www.phenoscanner.medschl.cam.ac.uk/)^[30]^ and searched for individual phenotypes corresponding to the IVs. We assessed whether these phenotypes were directly associated with the outcome. If they were, the corresponding rs numbers for the phenotypes were identified as confounding SNPs and subsequently excluded. Finally, we remove the SNPs with palindromic properties and incompatible alleles.

### 2.4. Mendelian randomization analysis

The standard inverse-variance weighted (IVW) method^[31]^ was used as the primary analysis to calculate the relationship between metformin and IMIDs. Furthermore, the MR-Egger, weighted median, simple mode, and weighted mode methods^[32,33]^ were conducted as supplementary analyses. Currently, IVW is the most widely used statistic in MR analysis, which assume in its simplest form that all genetic variants are effective IVs, providing estimates and precision similar to 2-stage least squares.^[34,35]^

### 2.5. Sensitivity analysis

In this study, we performed Cochran *Q* statistic to test heterogeneity.^[36]^ The *Q*-test *P* < .05 indicates the presence of heterogeneity, and a random-effects model was used to assess the results. The MR-Egger-intercept test and the outlier (MR-PRESSO) test were utilized to detect pleiotropy.^[37]^ When the intercept *P* > .05, it means that there is an absence of horizontal pleiotropic. Finally, leave-one-out analysis can determine whether any single SNPs are strongly driving causal associations.

### 2.6. Statistical analysis

All data analyses were conducted in R software (version 4.2.3) with the R packages “TwosampleMR (version 0.5.8).” The difference was considered statistically significant only if the *P*-value < .05. And the results of MR analyses were expressed as odds ratios (OR) with 95% confidence intervals.

## 3. Results

### 3.1. Selection of IVs

IVs associated with metformin (exposure) and IMIDs (outcome) were selected by using R software according to the screening criteria (40 SNPs for RA, 44 SNPs for CD, 45 SNPs for UC, 45 SNPs for SLE, 34 SNPs for MS, 45 SNPs for AIH, 30 SNPs for PBC, 39 SNPs for PSC). Finally, 39 SNPs were selected as IVs for RA due to 1 SNP (*rs11658063*) being palindromic. And the *F*-values of these IVs were above the threshold of 10, proving that the screened IVs did not show weak instrument bias.

### 3.2. Causal effect of metformin treatment on IMIDs

**Figure 2.**
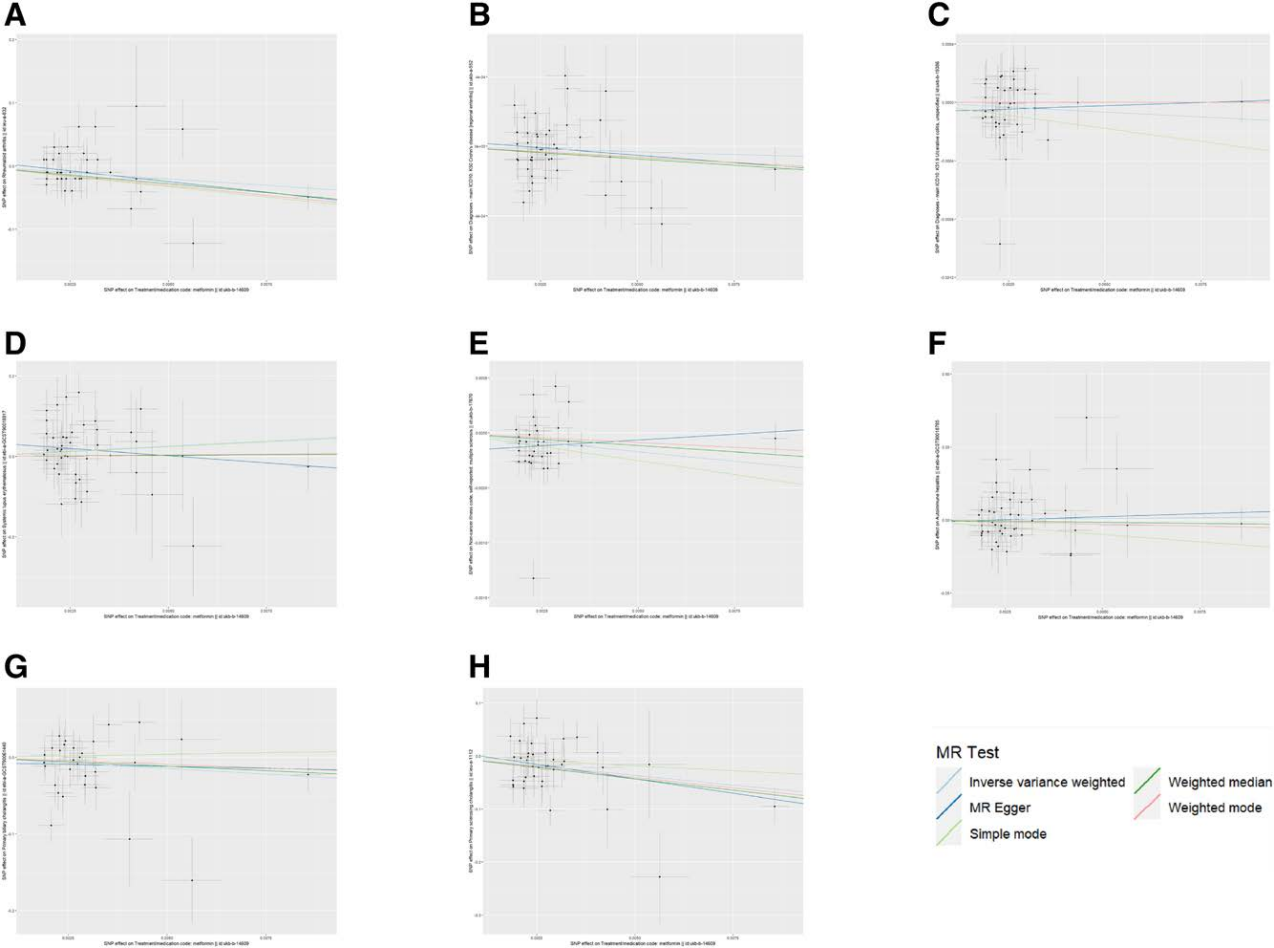
The scatter plots of MR analysis. (A) metformin on RA; (B) metformin on CD; (C) metformin on UC; (D) metformin on SLE; (E) metformin on MS; (F) metformin on AIH; (G) metformin on PBC; (H) metformin on PSC. AIH = autoimmune hepatitis, CD = Crohn disease, MR = Mendelian randomization, MS = multiple sclerosis, PBC = primary biliary cholangitis, PSC = primary sclerosing cholangitis, RA = rheumatoid arthritis, SLE = systemic lupus erythematosus, UC = ulcerative colitis.

We performed MR analysis on metformin with 8 IMIDs, including RA, CD, UC, SLE, MS, AIH, PBC, and PSC. The IVW analyses were used as the primary method. The results of MR analysis showed that the slopes of the solid lines in the scatter plot of the effects of genetic variants on metformin treatment and IMIDs indicate that the incidence of IMIDs is negatively correlated with metformin treatment. The IVW analysis showed that the treatment of metformin can significantly reduce the risk of RA (OR = 0.018, 95% CI: 1.33 × 10^−3^–0.233, *P* = .002), MS (OR = 0.966, 95% CI: 0.936–0.997, *P* = .030) and PSC (OR = 6.82 × 10^−4^, 95% CI: 7.83 × 10^−6^–5.93 × 10^−2^, *P* = .001). In addition, MR-Egger, weighted median and weighted mode also supported the results of causal associations between metformin and RA except for the simple mode. Between metformin and PSC, of the 4 additional methods carried out, weighted median and weighted mode also suggested significant difference. But for metformin with MS, the other 4 methods all did not support their causal associations. IVW also showed metformin treatment is not significantly associated with the risk of CD (OR = 0.994, 95% CI: 0.979–1.009, *P* = .431), UC (OR = 0.987, 95% CI: 0.965–1.009, *P* = .234), SLE (OR = 164.373, 95% CI: 0.158–1.71 × 10^5^, *P* = .150), AIH (OR = 2.909, 95% CI: 4.58 × 10^−3^–1.85 × 10^3^, *P* = .746) and PBC (OR = 0.055, 95% CI: 1.44 × 10^−3^–2.112, *P* = .119). Meanwhile, the MR-Egger, weighted mode, simple mode, and WM results were consistent with the IVW. The results of MR analysis as illustrated in Table 2. Figure 2 illustrates scatter plots depicting metformin to RA, CD, UC, SLE, MS, AIH, PBC, and PSC causal associations. The forest plots are shown in Figure S1, Supplemental Digital Content, http://links.lww.com/MD/O348. The detailed information of SNPs is listed in Tables S1–S12, Supplemental Digital Content, http://links.lww.com/MD/O347.

**Table 2:** The MR analysis results of exposure and outcomes.

Outcome	Method	SNPs (n)	*P*	OR	LCI	UCI
RA	MR-Egger	39	.036	1.14 × 10^−3^	2.54 × 10^−6^	5.16 × 10^−1^
	WM	39	.003	3.74 × 10^−3^	9.50 × 10^−5^	1.47 × 10^−1^
	IVW	39	.002	1.76 × 10^−2^	1.33 × 10^−3^	2.33 × 10^−1^
	Simple model	39	.069	1.49 × 10^−3^	1.61 × 10^−6^	1.38
	Weighted model	39	.003	2.15 × 10^−3^	4.55 × 10^−5^	1.01 × 10^−1^
CD	MR-Egger	44	.401	9.83 × 10^−1^	9.45 × 10^−1^	1.023
	WM	44	.201	9.86 × 10^−1^	9.64 × 10^−1^	1.009
	IVW	44	.431	9.94 × 10^−1^	9.79 × 10^−1^	1.009
	Simple model	44	.536	9.87 × 10^−1^	9.47 × 10^−1^	1.028
	Weighted model	44	.336	9.88 × 10^−1^	9.64 × 10^−1^	1.012
UC	MR-Egger	38	.766	1.009	9.53 × 10^−1^	1.068
	WM	38	.998	9.99 × 10^−1^	9.72 × 10^−1^	1.029
	IVW	38	.234	9.87 × 10^−1^	9.65 × 10^−1^	1.009
	Simple model	38	.153	9.65 × 10^−1^	9.20 × 10^−1^	1.012
	Weighted model	38	.999	1.000	9.68 × 10^−1^	1.033
UC (adjusted)	IVW	37	.355	9.92 × 10^−1^	9.75 × 10^−1^	1.009
SLE	MR-Egger	45	.468	7.93 × 10^−4^	4.03 × 10^−12^	1.56 × 10^5^
	WM	45	.899	1.799	1.90 × 10^−4^	1.71 × 10^4^
	IVW	45	.150	1.64 × 10^2^	1.58 × 10^−1^	1.71 × 10^5^
	Simple model	45	.643	1.25 × 10^2^	1.90 × 10^−7^	8.28 × 10^10^
	Weighted model	45	.903	2.231	5.46 × 10^−6^	9.12 × 10^5^
SLE (adjusted)	IVW	43	.409	1.44 × 10^1^	2.55 × 10^−2^	8.13 × 10^3^
MS	MR-Egger	34	.595	1.021	9.45 × 10^−1^	1.104
	WM	34	.083	9.77 × 10^−1^	9.52 × 10^−1^	1.003
	IVW	34	.030	9.66 × 10^−1^	9.36 × 10^−1^	9.97 × 10^−1^
	Simple model	34	.090	9.51 × 10^−1^	8.98 × 10^−1^	1.006
	Weighted model	34	.198	9.83 × 10^−1^	9.57 × 10^−1^	1.009
MS (adjusted)	IVW	29	8.76 × 10^−8^	9.46 × 10^−1^	9.27 × 10^−1^	9.65 × 10^−1^
AIH	MR-Egger	45	.646	6.30 × 10^1^	1.52 × 10^−6^	2.61 × 10^9^
	WM	45	.765	2.21 × 10^−1^	1.13 × 10^−5^	4.33 × 10^3^
	IVW	45	.746	2.909	4.58 × 10^−3^	1.85 × 10^3^
	Simple model	45	.259	4.90 × 10^−5^	1.97 × 10^−12^	1.22 × 10^3^
	Weighted model	45	.624	6.41 × 10^−2^	1.17 × 10^−6^	3.52 × 10^3^
PBC	MR-Egger	30	.818	3.61 × 10^−1^	6.52 × 10^−5^	1.99 × 10^3^
	WM	30	.294	9.76 × 10^−2^	1.27 × 10^−3^	7.524
	IVW	30	.119	5.52 × 10^−2^	1.44 × 10^−3^	2.112
	Simple model	30	.857	2.241	3.70 × 10^−4^	1.36 × 10^4^
	Weighted model	30	.432	1.58 × 10^−1^	1.66 × 10^−3^	1.49 × 10^1^
PBC (adjusted)	IVW	27	.132	1.06 × 10^−1^	5.73 × 10^−3^	1.969
PSC	MR-Egger	39	.061	1.87 × 10^−5^	3.03 × 10^−10^	1.149
	WM	39	.008	1.90 × 10^−4^	3.548 × 10^−7^	1.02 × 10^−1^
	IVW	39	.001	6.82 × 10^−4^	7.834 × 10^−6^	5.93 × 10^−2^
	Simple model	39	.601	2.48 × 10^−2^	2.656 × 10^−8^	2.31 × 10^4^
	Weighted model	39	.033	3.38 × 10^−4^	2.899 × 10^−7^	3.95 × 10^−1^

Abbreviations: AIH = Autoimmune hepatitis, CD = Crohn disease, IVW = inverse-variance weighted, LCI = lower confidence interval, MR = Mendelian randomization, MS = multiple sclerosis, OR = odds ratio, PBC = Primary biliary cholangitis, PSC = Primary sclerosing cholangitis, RA = rheumatoid arthritis, SLE = Systemic lupus erythematosus, SNPs = single-nucleotide polymorphisms, UC = ulcerative colitis, UCI = upper confidence interval, WM = weight median.

### 3.3. Sensitivity analysis

The results of Cochran *Q*-test for heterogeneity are presented in Table 3. The analysis revealed significant heterogeneity of metformin to UC (*p*_*IVW*_ = 0.005), SLE (*p*_*IVW*_ = 0.035), MS (*p*_*IVW*_ < 0.001), and PBC (*p*_*IVW*_ = 0.010). The other groups exhibited no heterogeneity (*P* > .05). Due to the heterogeneity observed in these 4 causality pairs, SNPs with significant differences were excluded through outlier testing. Specifically, 1, 2, 5, and 3 SNPs were removed for the UC, SLE, MS, and PBC groups, respectively. We performed MR analysis again after removing these SNPs with significant differences. The results indicated that metformin treatment significantly reduces the risk of MS (OR = 0.946, 95% CI: 0.927–0.965, *P* < .001). The results also suggested that metformin treatment is not significantly associated with the risk of UC (OR = 0.946, 95% CI: 0.927–0.965, *P* = .355), SLE (OR = 14.407, 95% CI: 0.026–8.13 × 10^3^, *P* = .409) and PBC OR = 0.106, 95% CI: 5.73 × 10^−3^–1.969, *P* = .132). The adjusted scatter and forest plots are shown in Figure S2, Supplemental Digital Content, http://links.lww.com/MD/O348. After adjustment, Cochran *Q* analysis revealed no significant heterogeneity of metformin to UC (*P* = .610), SLE (*P* = .262), MS (*P* = .776), and PBC (*P* = .574). Meanwhile, in the analysis results of MR-PRESSO, it was found that there was absence of genetic level pleiotropy (Table 3). The symmetry of the funnel plot indicated no publication bias (Figure S3, Supplemental Digital Content, http://links.lww.com/MD/O348). Additionally, leave-one-out sensitivity testing demonstrated that the causal effect of metformin treatment on IMIDs was not significantly influenced by the exclusion of any single SNP (Figure S4, Supplemental Digital Content, http://links.lww.com/MD/O348). These results can be shown to be stable and reliable.

**Table 3:** Sensitivity analyses of the causal effect of metformin treatment on IMIDs.

Outcome	Method	SNPs (n)	Cochran *Q*-test			Pleiotropy test		
			*Q*	*Q*-df	*P*	Egger-intercept	SE	*P*
RA	MR-Egger	39	50.75	37	.066	−9.35 × 10^−3^	9.67 × 10^−3^	.339
	IVW		52.03	38	.064			
CD	MR-Egger	44	55.99	42	.073	−3.47 × 10^−5^	5.89 × 10^−5^	.559
	IVW		56.46	43	.082			
UC	MR-Egger	38	61.51	36	.005	−6.75 × 10^−5^	8.22 × 10^−5^	.417
	IVW		62.66	37	.005			
UC adjusted	MR-Egger	37	32.71	35	.579	−3.58 × 10^−5^	6.31 × 10^−5^	.574
	IVW		33.04	36	.610			
SLE	MR-Egger	45	59.84	43	.045	3.76 × 10^−2^	2.79 × 10^−2^	.185
	IVW		62.36	44	.035			
SLE adjusted	MR-Egger	43	45.95	41	.275	2.85 × 10^−2^	2.52 × 10^−2^	.264
	IVW		47.39	42	.262			
MS	MR-Egger	34	113.93	32	4.05 × 10^−11^	−1.70 × 10^−4^	1.10 × 10^−4^	.134
	IVW		122.33	33	3.43 × 10^−12^			
MS adjusted	MR-Egger	29	21.87	27	.744	−7.02 × 10^−5^	1.45 × 10^−4^	.631
	IVW		22.10	28	.776			
AIH	MR-Egger	45	54.26	43	.117	−9.57 × 10^−3^	2.58 × 10^−2^	.713
	IVW		54.43	44	.135			
PBC	MR-Egger	30	49.10	28	.008	−6.62 × 10^−3^	1.40 × 10^−2^	.640
	IVW		49.49	29	.010			
PBC adjusted	MR-Egger	27	24.04	25	.517	−3.25 × 10^−5^	1.09 × 10^−2^	.998
	IVW		24.04	26	.574			
PSC	MR-Egger	39	50.35	37	.070	1.16 × 10^-2^	1.66 × 10^-2^	.488
	IVW		51.02	38	.077			

Abbreviations: AIH = autoimmune hepatitis, CD = Crohn disease, IMIDs = immune-mediated inflammatory diseases, IVW = inverse-variance weighted, MR = Mendelian randomization, MS = multiple sclerosis, PBC = primary biliary cholangitis, PSC = primary sclerosing cholangitis, RA = rheumatoid arthritis, SE = standard error, SLE = systemic lupus erythematosus, SNPs = single-nucleotide polymorphisms, UC = ulcerative colitis.

## 4. Discussion

Although metformin has been used as a therapeutic agent for T2DM for decades, its molecular mechanisms remain under investigation. The hypoglycemic effects of metformin involve multiple mechanisms.^[5]^ First, metformin acts on Adenosine 5‘-monophosphate-activated protein kinase (AMPK) to lower hepatic glucose production and reduce peripheral blood glucose concentration.^[38]^ Second, metformin induces hepatocyte AMPK activation, leading to the downregulation of glycogen gene transcription.^[39]^ Metformin also acts on complex 1 in mitochondria to promote AMPK activation.^[40]^ In addition to this, metformin regulates blood glucose levels by inhibiting gluconeogenic enzymes, glucagon signaling, and mitochondrial enzymes.^[41–43]^ In recent years, many studies have found that metformin also has anti-inflammatory and immunomodulatory effects.^[44]^ Metformin can inhibit the JAK-STAT pathway directly or indirectly via AMPK, limiting interleukin 6 (IL-6)-mediated inflammatory responses.^[45]^ And the STAT3 signaling pathway is required not only for TH17 cell differentiation but also for T follicular helper (TFH), memory B, plasma cell differentiation and antigen-specific antibodies responses.^[46]^ Furthermore, AMPK-dependent inhibition of STAT3 and nuclear factor kappa-light-chain-enhancer of activated B cells (NF-κBs) pathways are involved in the inhibition of monocyte-to-macrophage differentiation, and reduce the production of inflammatory cytokines by macrophages.^[47,48]^ Therefore, metformin becomes a possibility to treat and prevent autoimmune and inflammatory diseases by regulating multiple cells signaling pathways through AMPK. The abnormal activation of the immune system and inflammatory pathways leads to the systemic involvement of IMIDs.

In this study, we identified a causal effect of metformin treatment on some IMIDs. The results suggested that metformin treatment reduces the risk of RA, MS, and PSC. Although results have indicated that metformin does not significantly decrease the incidence of UC, CD, SLE, AIH, and PBC, it appears that metformin treatment is negatively correlated with the risk of their development, with a tendency to reduce the incidence of these diseases. At present, many articles have shown that metformin treatment can reduce inflammation, delay disease progression and adjust immune system function. But few studies focus on the relationship between metformin and the risk of IMIDs, and these studies are controversial. Naffaa^[49]^ and Seoyoung^[50]^ found metformin therapy can reduced the risk of RA in women through Dipeptidyl peptidase-4 (DPP4). However, Zemedikun^[17]^ and Lu^[51]^ did not agree with this view. For IBD, there is also no unanimity. In a retrospective study of Taiwan, researchers found the risk of IBD was reduced by half in patients treated with metformin compared to patients treated with other antidiabetic agents.^[52]^ However, the results of a Danish study showed no association between the history or duration of metformin use and the risk of IBD.^[53]^ These conflicting results may be related to racial and geographic differences on the 1 hand, and partly due to the bias of retrospective studies on the other. In practice, metformin also may cause side effects, such as acidosis, nausea, abdominal discomfort and diarrhea.^[54]^ But a randomized trial has confirmed metformin is safe for nondiabetic persons.^[55]^

This study is based on a large-scale MR analysis from GWAS summary statistics, circumventing traditional confounding factors and problems associated with reverse causation. Then the *F*-statistics of all IVs > 10 avoided potential weak instrumental bias. Additionally, several sensitivity tests were conducted to ensure the stability of the results. However, our study does have some limitations. All participants included in the research were of European, which limits the generalizability of our findings. Furthermore, the lack of demographic data in the original study hindered further analyses. Lastly, since the exposure factor in this study was a drug treatment, we were unable to perform bidirectional MR analyses.

## 5. Conclusion

This study indicated that metformin therapy may reduce the risk of RA, MS, and PSC in European populations, providing new genetic evidence for the relationship between metformin and some IMIDs. Although metformin does not significantly decrease the risk of the other 5 diseases examined, there is a discernible trend indicating that metformin may lower their incidence. The mechanisms by which metformin inhibits IMIDs remain unclear and are a subject of ongoing debate. Further clinical studies and experimental research are necessary to validate the role of metformin in the prevention of IMIDs.

## 6. Other information

We guarantee that our manuscript is a unique submission and will not be considered for publication in any media by any other source. Furthermore, the manuscript has not been published, in part or in full, in any form. All authors have read and approved the final manuscript and agree to take responsibility for all aspects of the work. All authors have no funding and conflicts of interest to disclose. The datasets generated during and/or analyzed during the current study are publicly available. The summary GWAS data used in this study are publicly available and no specific ethical approval was required. Supplemental Digital Content is available for this article.

## Author contributions

**Data curation:** Zheng Liao.

**Project administration:** Jinlong Liu.

**Software:** Zheng Liao.

**Supervision:** Jian Li.

**Validation:** Chenguang Su.

**Visualization:** Chenguang Su.

**Writing – original draft:** Zheng Liao, Chenguang Su.

**Writing – review & editing:** Jian Li, Jinlong Liu.

## Supplementary Material


