# Supplementary material for: Causal association of metformin treatment with diverse immune-mediated inflammatory diseases: A Mendelian randomization analysis
Source: Medicine (Baltimore). 2025 Feb 7;104(6):e41400. doi: 10.1097/MD.0000000000041400 (PMC11813035; doi:10.1097/MD.0000000000041400)
Supplement: Supplementary file 2 [file medi-104-e41400-s002.pdf]

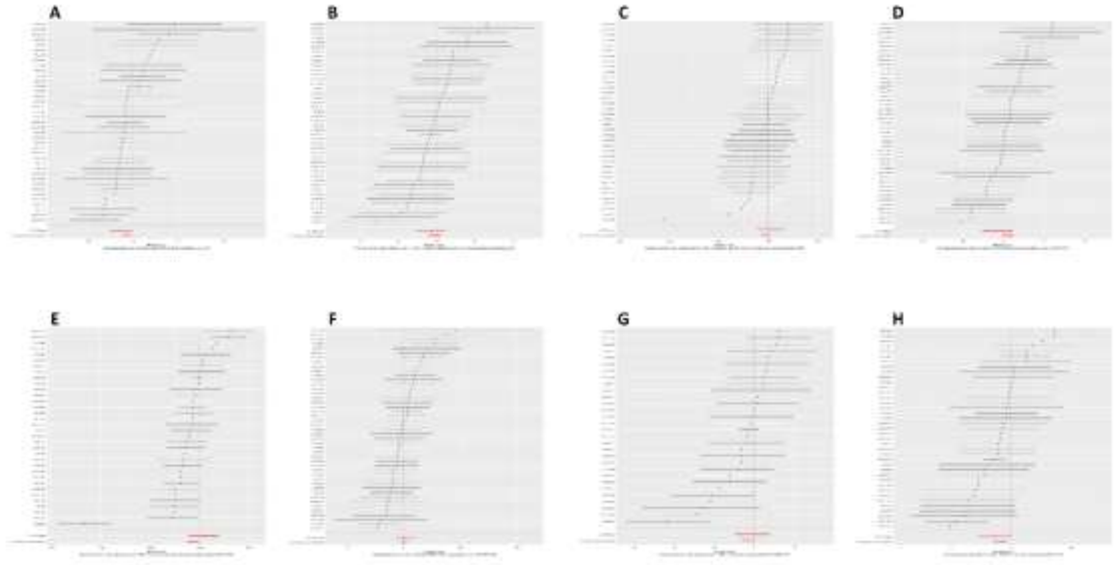

Supplementary Figure 1. *The forest plots of MR analysis. (A) metformin on RA; (B) metformin on CD; (C) metformin on UC; (D) metformin on SLE; (E) metformin on MS; (F) metformin on AIH; (G) metformin on PBC; (H) metformin on PSC. RA, rheumatoid arthritis; CD, Crohn's disease; UC, ulcerative colitis; SLE, Systemic lupus erythematosus; MS, multiple sclerosis; AIH, Autoimmune hepatitis; PBC, Primary biliary cholangitis; PSC, Primary sclerosing cholangitis.*

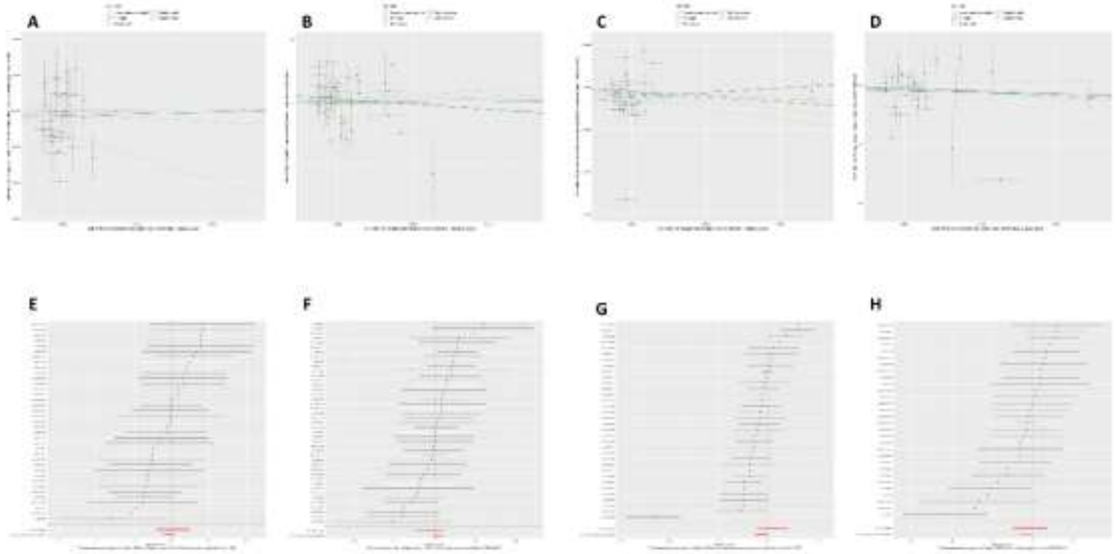

Supplementary Figure 2. *The scatter and forest plots of MR analysis. (A) (E) metformin on UC; (B) (F) metformin on SLE; (C) (G) metformin on MS; (D) (H) metformin on PBC. UC, ulcerative colitis; SLE, Systemic lupus erythematosus; MS, multiple sclerosis; PBC, Primary biliary cholangitis.*

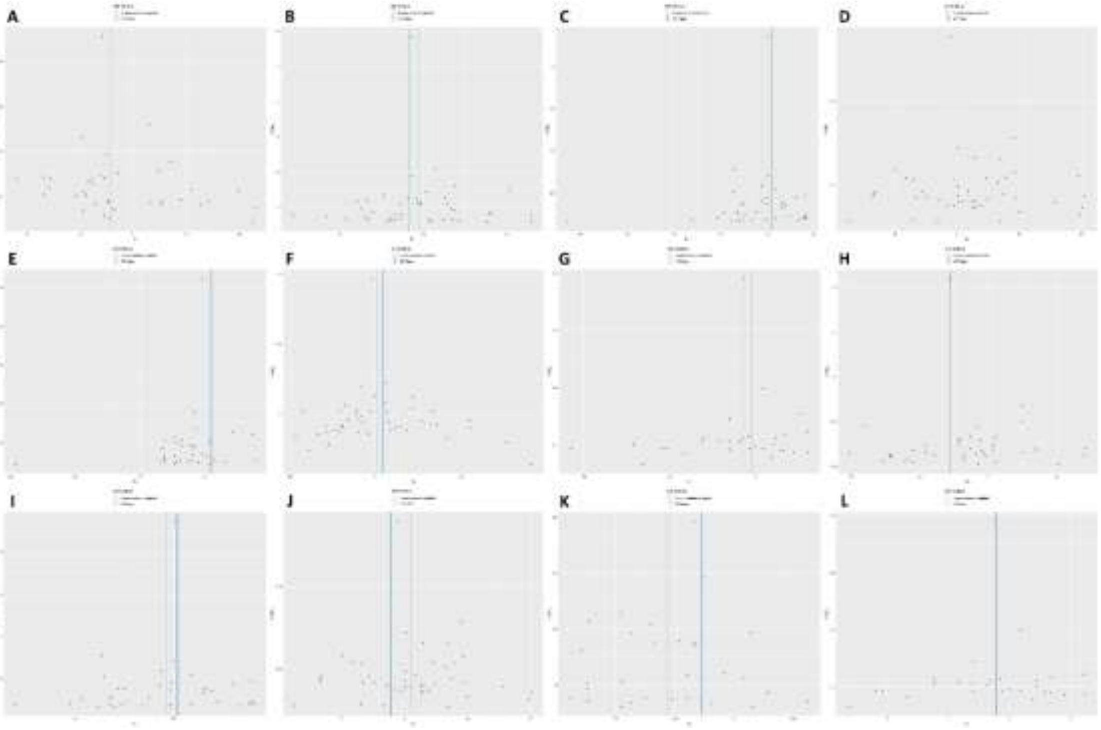

Supplementary Figure 3. *The funnel plots of MR analysis. (A) metformin on RA; (B) metformin on CD; (C) metformin on UC; (D) metformin on SLE; (E) metformin on MS; (F) metformin on AIH; (G) metformin on PBC; (H) metformin on PSC; (I) metformin on UC after adjustment; (J) metformin on SLE after adjustment; (K) metformin on MS after adjustment; (L) metformin on PBC after adjustment. RA, rheumatoid arthritis; CD, Crohn's disease; UC, ulcerative colitis; SLE, Systemic lupus erythematosus; MS, multiple sclerosis; AIH, Autoimmune hepatitis; PBC, Primary biliary cholangitis; PSC, Primary sclerosing cholangitis.*

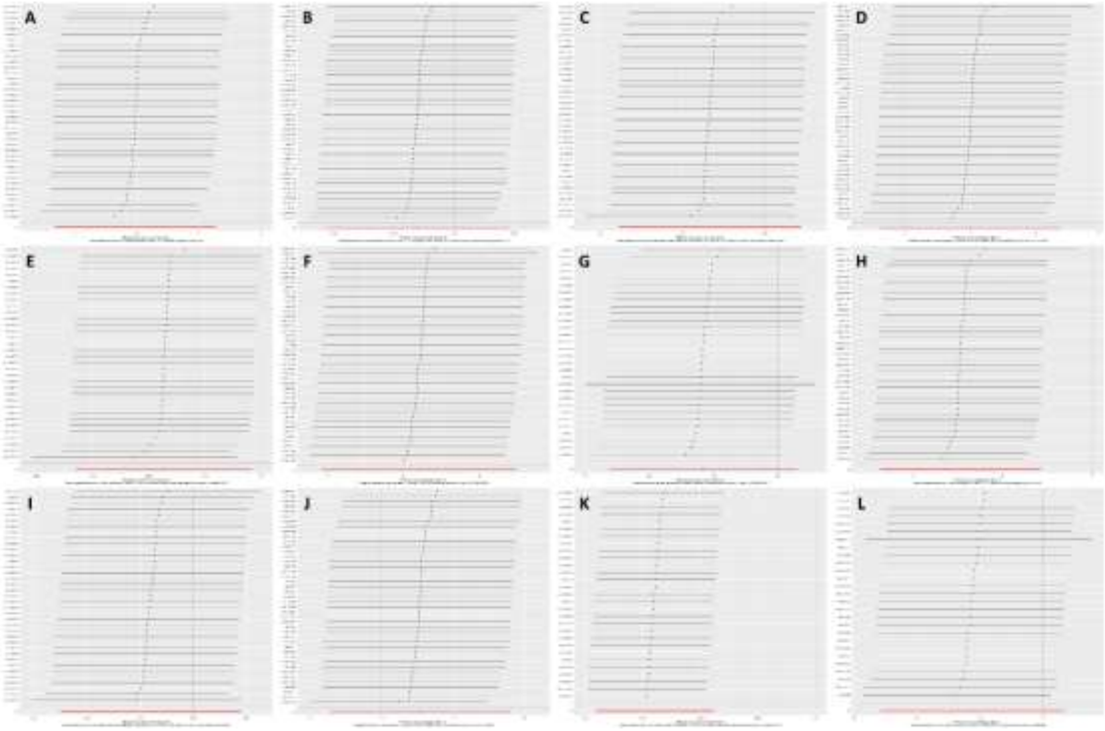

Supplementary Figure 4. *MR leave-one-out sensitivity analysis of MR analysis. (A) metformin on RA; (B) metformin on CD; (C) metformin on UC; (D) metformin on SLE; (E) metformin on MS; (F) metformin on AIH; (G) metformin on PBC; (H) metformin on PSC; (I) metformin on UC after adjustment; (J) metformin on SLE after adjustment; (K) metformin on MS after adjustment; (L) metformin on PBC after adjustment. RA, rheumatoid arthritis; CD, Crohn's disease; UC, ulcerative colitis; SLE, Systemic lupus erythematosus; MS, multiple sclerosis; AIH, Autoimmune hepatitis; PBC, Primary biliary cholangitis; PSC, Primary sclerosing cholangitis.*
